# Proposed diagnostic and prognostic markers of primary malignant hepatic vascular neoplasms

**DOI:** 10.1186/s13000-024-01482-5

**Published:** 2024-05-13

**Authors:** Youngeun Yoo, Jinho Shin, Eunsung Jun, Eun-Young Koh, Hwa Jeong Shin, Hyo Jeong Kang

**Affiliations:** 1 Department of Pathology, Asan Medical Center, University of Ulsan College of Medicine, 88 Olympic-ro 43-gil, Songpa-gu, Seoul, 05505 Republic of Korea; 2 Department of Medicine, University of Ulsan College of Medicine, Seoul, Republic of Korea; 3 Department of Convergence Medicine, Asan Medical Center, Asan Institute for Life Sciences, University of Ulsan College of Medicine Seoul, Seoul, Republic of Korea; 4 Department of Research Support Team, Asan Medical Center, University of Ulsan College of Medicine, Seoul, Republic of Korea; 5 Asan Liver Center, Asan Medical Center, University of Ulsan College of Medicine, Seoul, Republic of Korea

**Keywords:** Epithelioid hemangioendothelioma, Angiosarcoma, Liver, Vascular neoplasm, Malignancy, Diagnosis

## Abstract

**Introduction:**

Primary malignant hepatic vascular tumors with various malignant potentials include epithelioid hemangioendothelioma (EHE) and angiosarcoma (AS), which may overlap pathologically. This study aimed to compare the pathological findings of hepatic EHE with those of AS, in association with patient outcomes.

**Methods:**

Fifty-nine histologically confirmed patients with 34 EHE and 25 AS were admitted to a tertiary hospital from 2003 to 2020. Their EHE and AS pathological features were compared. Immunohistochemistry for CD31, ERG, CAMTA-1, TFE3, P53, and Ki-67 labeling was performed on paraffin-embedded blocks. Markers, along with histological findings, were analyzed for the purposes of diagnostic and prognostic significance by multivariate analysis.

**Results:**

CAMTA-1 was 91.2% positive in EHE, but negative in AS (*p = <* 0.001). AS was significantly correlated to an aberrant p53 expression, high Ki-67 labeling, and high mitotic activity, compared to EHE (all, *p = <* 0.001). EHE can be classified as low grade (LG) and high grade (HG) using the prognostic values of mitotic activity and ki-67 labeling (sensitivity = 1, specificity = 1). Low grade-EHE showed significantly better overall survival than high grade-EHE (*p* = 0.020).

**Conclusions:**

Immunohistochemistry for CAMTA-1, P53, and Ki-67 labeling may help distinguish EHE and AS in histologically ambiguous cases, especially small biopsied tissue. Moreover, the combination of mitotic activity and Ki-67 labeling can be a prognostic factor for EHE with various clinical features.

## Introduction

Hepatic malignant vascular tumors include a wide range of malignancies – hepatic epithelioid hemangioendothelioma (EHE) with low-to-intermediate grade malignancy, and angiosarcoma (AS), which is highly malignant with a negative prognosis [1–4]. While AS is invariably aggressive with a high rate of local recurrence and metastatic potential, the progress of EHE is heterogeneous, ranging from indolent to aggressive. Given the differences in treatment and prognosis between EHE and AS, it is important to differentiate between these two tumors [5, 6].

Both EHE and AS usually manifest as multiple hepatic lesions with some confusing and complex imaging features [7–9]. Due to the overlapping imaging features of EHE and AS, a liver biopsy is not infrequently required. However, the pathological findings of EHE and AS may overlap, which makes it hard to suggest a definite diagnosis even with a liver biopsy, especially if only small biopsy specimens are obtained, or there is no apparent endothelial differentiation. Recently, nuclear Calmodulin Binding Transcription Activator 1 (CAMTA1) expression has been suggested as a useful marker for EHE diagnosis [10, 11]. However, previous studies included a limited number of patients. In addition, there are no internationally recognized pathological criteria to assist with the prediction of the course of EHE in terms of various clinical outcomes.

This study aimed to evaluate the pathological findings of EHE and AS with different malignant potentials in correlation with patient outcomes. Furthermore, we suggest diagnostic markers to distinguish these tumors accurately and present pathological guidelines for predicting prognoses.

## Materials and methods

### Patients and samples

**Fig. 1:**
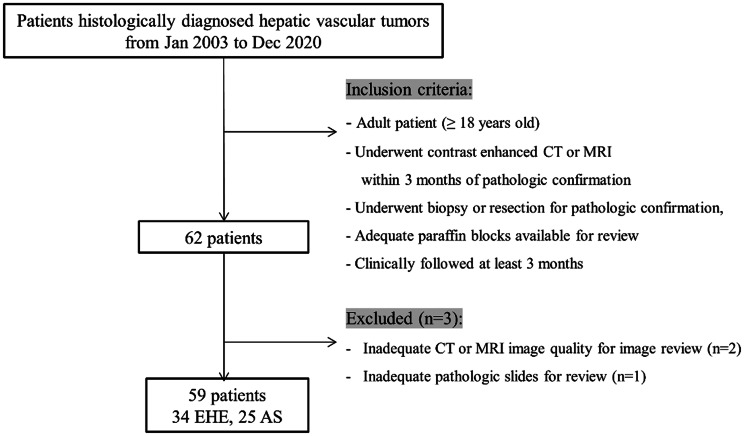
Flow diagram showing exclusion criteria for the selection of EHE and AS. EHE: epithelioid hemangioendothelioma; AS: angiosarcoma

This single-center retrospective study was approved by the Institutional Review Board of Asan Medical Center, Seoul, Korea (approval No. 2021 − 0766). An electronic data search in our pathologic database identified 59 cases of histologically diagnosed hepatic vascular tumors including EHE and AS between January 2003 and December 2020. The inclusion criteria were as follows: (a) adult patient (≥ 18 years old); (b) who underwent contrast-enhanced Computed Tomography (CT) or Magnetic Resonance Imaging (MRI) within three months of pathologic confirmation; (c) who underwent biopsy or resection for pathologic confirmation; (d) who had adequate paraffin blocks available for review; and (e) who had been clinically followed at least three months after the pathologic confirmation. We excluded patients who had inadequate CT or MRI image quality for imaging review, and those without adequate pathologic slides for review (Fig. 1). Finally, 59 patients (34 EHE, and 25 AS) were included in this study.

### Clinical information

Clinical information, including age at biopsy or surgical resection; sex; most recent lab data after pathologic confirmation; presence of the hepatitis virus; serum alanine aminotransferase (ALT); aspartate aminotransferase (AST); alkaline phosphatase (ALP); gamma-glutamyltransferase (GGT); total bilirubin, alpha-fetoprotein (AFP) level; albumin; platelet; prothrombin time (PT); protein induced by vitamin K absence or antagonists II (PIVKA II); and date of disease recurrence and death or last follow-up, were obtained from electronic medical records.

### Pathologic assessment

All available hematoxylin and eosin (H&E)-stained slides were reviewed without knowledge of the clinical information and pathological characteristics. One representative paraffin block to be used for immunohistochemistry was selected for each case after a close pathologic review by two pathologists (H.J.K. and J.H.S). Additional histopathological features were evaluated as follows; tumor border, tumor size (cm), tumor number, hemorrhage, necrosis, solid/sheet growth, myxohyaline stroma, sinusoidal infiltration, hypercellularity, buds, hobnails or papillary-like projections, high-grade nuclear atypia, prominent nucleoli, cytoplasmic vacuoles, and the number of mitotic figures per 10 high-power fields. The area of 10 HPFs reaches 2mm^2^.

### Immunohistochemical staining and evaluation

Immunohistochemistry (IHC) was performed using a Benchmark XT (Ventana Medical Systems, Tucson, AZ) autoimmunostainer with an Optview DAB IHC detection kit (Ventana Medical Systems) according to the manufacturer’s instructions. Briefly, 4-µm-thick sections of representative formalin-fixed paraffin-embedded tissue blocks were deparaffinized and rehydrated by immersion in xylene and a graded ethanol series. Endogenous peroxidase was blocked by incubation in 3% H_2_O_2_ for 10 min, followed by heat-induced antigen retrieval. The sections were incubated at room temperature for 32 min in primary antibodies for CD 31 (1:500, Mouse monoclonal, clone JC70, CELL MARQUE, Rocklin, CA, USA), ERG (1:400, Rabbit monoclonal, clone EP111, NEOMARKERS, Rocklin, CA, USA), CAMTA1 (1:200, Rabbit polyclonal, NOVUS, CO, USA), TFE3 (1:100, Rabbit monoclonal, clone MRQ-37, CELL MARQUE, Rocklin, CA, USA), KI-67 (1:200, Mouse monoclonal, clone MIB1, DAKO, Glostrup, Denmark), and P53 (1:1000, Mouse monoclonal, clone DO-7, DAKO, Glostrup, Denmark). Immunostained sections were lightly counterstained with hematoxylin, dehydrated in ethanol, and cleared in xylene.

Immunolabeled slides were independently evaluated by two experienced pathologists (H.J.K. and J.H.S). The expression of CD31, ERG, and CAMTA1 was evaluated as “negative” and “positive” regardless of intensity and proportion. The expression of TFE3 and P53 was scored according to the proportion and classified under the following criteria; score 0: no expression, or < 10% staining, score 1: 10% to 1/3rd part staining, score 2: 1/3 to 2/3rd part staining, score 3: >2/3rd part staining. A TFE3 and P53-nuclear staining score of 3 was considered positive. Immunostaining for p53 has been used as a surrogate marker for the presence of a *TP53* mutation. An aberrant p53 expression, associated with a *TP53* mutation was defined as the form of strong diffuse nuclear positivity or null-type pattern.

### Quantitative evaluation of Ki-67 labeling index

For quantitative analysis of Ki-67 labeling positivity in neoplastic cells, digital slide images were generated with a Pannoramic 250 Flash III (3DHistech, Hungary), and ERG and Ki-67 IHC staining slides were analyzed with an open-source bioimage analysis software platform QuPath v0.3.0 [12]. After accommodating the 3,3’-diaminobenzidine staining vector with the “Estimating stain vectors” command, we counted Ki-67 positive cells with the “Positive cell detection” command, and Ki-67 positivity was calculated and used as the Ki-67 labeling index of the tumors. The cut-off point for a high Ki-67 was set to 10%.

### Statistical analysis

Clinical and pathological features of EHE and AS were compared according to the final pathologic diagnoses by means of the *t*-test or the Wilcoxon rank sum test for continuous variables and Fisher’s exact test for categorical variables. For imaging analysis, per patient analysis and per lesion analysis of imaging findings between EHE and AS were compared using consensually agreed imaging findings.

Overall survival (OS) was defined as the period from the initial pathologic diagnosis to death from any cause or last follow-up. OS times were calculated using the Kaplan–Meier method, and statistical significance was evaluated by the log-rank test. EHE were subdivided into low grade-EHE (LG EHE) and high grade-EHE (HG EHE) EHE according to different overall survival rates.

Statistical analyses were performed using the SPSS statistical software program (version 18.0 SPSS Inc. Chicago, IL, USA), and R (version 4.0.0).

## Results

### Patient characteristics

The clinical, imaging, and pathological characteristics of 59 patients are shown in Table 1. Patients with EHE (mean 49.6) were significantly younger than those with AS (mean 61.7). Females predominated the EHE group compared to the AS group (61.8% vs. 20%, *p* = 0.004). In the background of the liver, cirrhosis was observed exclusively in the AS group (70.8%, *p* = < 0.001). The AS group showed significantly lower levels of albumin and platelets than the EHE group (both *p* = < 0.001). There were no differences between the two groups in the other serum liver function tests, tumor markers, and hepatitis virus status. In outcome data, deaths were observed more frequently in AS than in EHE (100% vs. 23.5%, *p* = < 0.001). All of the patients with AS died from the disease. The follow-up period (months) was shorter in AS than in EHE (10.2 ± 11.7 vs. 65.6 ± 52.3, *p* = < 0.001).

**Table 1:** Clinical and outcome information of 59 patients with EHE and AS

	EHE	AS	*p*-value
	(n = 34)	(n = 25)	
Clinical Information			
Age (years)	49.6 ± 13.2	61.7 ± 9.6	< 0.001*
Gender (Male : Female )	13 : 21	20 : 5	0.001*
Male	13 (38.2%)	20 (80.0%)	
Female	21 (61.8%)	5 (20.0%)	
Cirrhosis	0	17 (70.8%)	< 0.001*
Lab information			
ALT	32.7 ± 54.0	39.2 ± 39.8	0.610
AST	43.8 ± 95.0	72.0 ± 128.6	0.337
ALP	158.9 ± 372.6	163.2 ± 123.0	0.956
GGT	76.5 ± 133.8	132.4 ± 140.0	0.137
Albumin	3.9 ± 0.6	3.3 ± 0.6	< 0.001*
Prothrombin time	12.0 ± 1.5	16.9 ± 16.5	0.159
Total bilirubin	1.5 ± 3.5	3.4 ± 5.4	0.133
Platelet	244.4 ± 86.0	153.0 ± 101.6	< 0.001*
AFP	2.7 ± 1.4	3.4 ± 2.2	0.174
PIVKAII	25.1 ± 10.8	49.5 ± 92.9	0.270
HBV or HCV infection	5 (14.7%)	3 (12%)	0.199
Outcome information			
Follow-up period (months)	65.6 ± 52.3	10.2 ± 11.7	< 0.001*
Death	8 (23.5%)	25 (100%)	< 0.001*

* *p*-value < 0.05; EHE: epithelioid hemangioendothelioma; AS: angiosarcoma; ALT: serum alanine aminotransferase; AST: aspartate aminotransferase; ALP: alkaline phosphatase; GGT: gamma-glutamyltransferase; AFP: alpha-fetoprotein; PIVKAII: protein induced by vitamin K absence or antagonists II

### Comparison of histologic features between EHE and AS

**Fig. 2:**
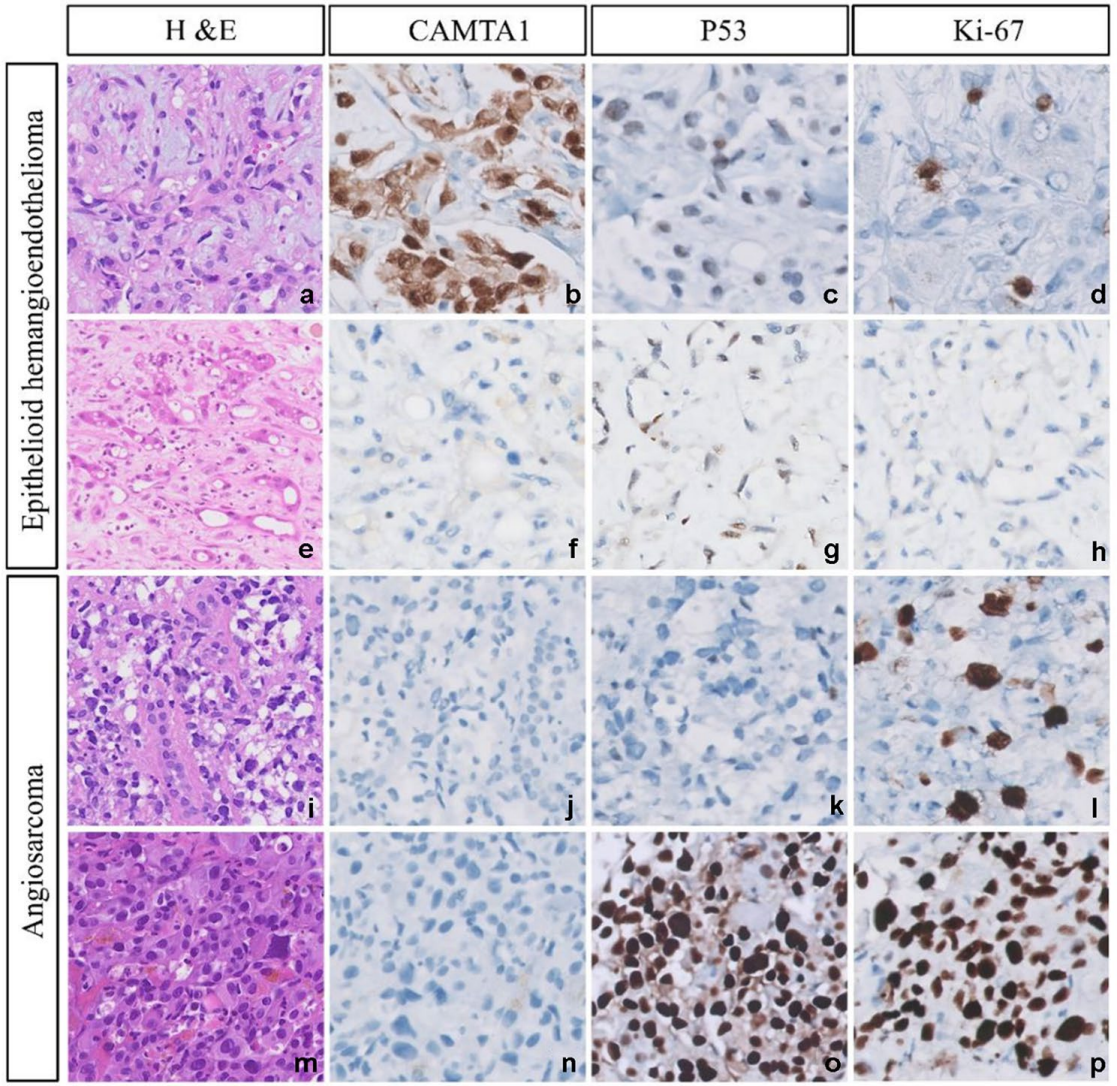
The histomorphology and immunohistochemical findings of EHE (**A**–**H**) and AS (I–P). EHE is characterized by myxohyaline stroma and cytoplasmic vacuoles in the H&E slide (**A** and **E**). A nuclear CAMTA1 expression is identified in most of the EHE (**B**), but it was negative in 10% (**F**). An aberrant p53 expression (**C** and **G**) and Ki-67 labeling index (**D** and **H**) are significantly lower in EHE. AS manifests with high cellularity and prominent nuclear atypia in the H&E slide (**I** and **M**). CAMTA1 is negative in all cases (**J** and **N**). AS shows aberrant p53 expressions (**K** and **L**) and high Ki-67 labeling indices (**O**, and **P**) (original magnification 200×, **A**–**P**). EHE: epithelioid hemangioendothelioma; AS: angiosarcoma

Histological characteristics between EHE and AS are summarized in Table 2; Fig. 2. The p-value was determined by multiple comparisons. Compared with AS, EHE was characterized by myxohyaline stroma (97.1%, *p* = < 0.001), buds, hobnails, or papillary-like projections (82.4%, *p* = < 0.001), and intracytoplasmic vacuoles (97.1%, *p* = < 0.001). Mitotic counts in EHE (1.26 ± 1.4/10HPFs) were significantly lower than in AS. In contrary, AS revealed general histopathological features including infiltrative tumor borders (100%, *p* = < 0.001), hemorrhage (40%, *p* = 0.004), solid/sheet growth (44%, *p* = < 0.001), sinusoidal infiltration (100%, *p* = 0.007), hypercellularity (44%, *p* = < 0.001), high grade nuclear atypia (96%, *p* = < 0.001), and high mitotic counts (mean ± SD/10HPFs, 9.66 ± 7.83/10HPFs). There were no significant differences between the two groups in tumor size (cm) (*p* = 0.052), tumor number (*p* = 0.067), necrosis (*p* = 0.160) and prominent nucleoli (*p* = 0.122).

**Table 2:** Comparison of histomorphology findings between EHE and AS

	EHE (n = 34)			AS (n = 25)			*p*-value
	N		%	N		%	
Border							< 0.001*
Well-defined	12	35.30%		0	0.00%		
Infiltrative	22	64.70%		25	100.00%		
Tumor size (cm)							0.052
<5 cm	21		61.80% 5	9		36.00%	
≥5 cm	13		38.20%	16		64.00%	
Tumor number							0.067
Simple	3	8.80%		4	16.00%		
Multiple (≥ 2)	31	91.20%		21	84.00%		
Hemorrhage							0.004*
Absent	31	91.20%		15	60.00%		
Present	3	8.80%		10	40.00%		
Necrosis							0.160
Absent	25	73.50%		14	56.00%		
Present	9	26.50%		11	44.00%		
Solid/sheet growth							< 0.001*
Absent	33	97.10%		14	56.00%		
Present	1	2.90%		11	44.00%		
Myxohyaline stroma							< 0.001*
Absent	1	2.90%		18	72.00%		
Present	33	97.10%		7	28.00%		
Sinusoidal infiltration							0.007*
Absent	9	26.50%		0	0.00%		
Present	25	42.40%		25	100.00%		
Hypercellularity							< 0.001*
Absent	34	57.60%		13	34.20%		
Present	0	0.00%		12	44.40%		
Buds, hobnails, or papillary-like projection							< 0.001*
Absent	6	17.60%		15	60.00%		
Present	28	82.40%		10	40.00%		
High grade nuclear atypia							< 0.001*
Absent	17	50.00%		1	4.00%		
Present	17	50.00%		24	96.00%		
Prominent nucleoli							0.122
Absent	27	79.40%		24	96.00%		
Present	7	20.60%		1	4.00%		
Cytoplasmic vacuoles							< 0.001*
Absent	1	2.90%		17	68.00%		
Present	33	97.10%		8	32.00%		
Mitosis/10HPFs							< 0.001*
Mean ± SD	1.26 ± 1.4 1.4			9.66 ± 7. 83 7.83			

* *p*-value < 0.05; EHE: epithelioid hemangioendothelioma; AS: angiosarcoma; SD: standard deviation

### IHC panel results

Immunohistochemically, ERG or CD31 expression was observed in all 59 cases (Table 3). The p-value was determined by multiple comparisons. CAMTA-1 nuclear positivity was observed in 31 of the 34 cases of EHE (91.2%). However, none of AS showed CAMTA-1 immunopositivity (*p* = < 0.001). Of the three cases that were negative for CAMTA-1, two cases were strongly positive for TFE3. TFE3 positivity was found in nine cases of EHE and in one case of AS (26.5% vs. 4.3%, *p* = 0.038). Ki-67 labeling index counted by QuPath was significantly higher in AS (mean 42.0%, range 12.6–69.5%) than in EHE (mean 6.0%, range 0.1–15.7%, *p* = < 0.001). Immunohistochemistry was not available for two cases of AS, but high Ki-67 (≥ 10%) was observed in all remaining AS. Additionally, immunostaining for p53 was performed as a surrogate marker for the presence of a *TP53* mutation. Aberrant p53 expression was more frequently identified in AS than in EHE (87% vs. 3%, *p* = < 0.001) (Fig. 2).

**Table 3:** Comparison of immunohistochemical results between EHE and AS

	EHE (n = 34)		AS (n = 25)		*p*-value
	N	(%)	N	(%)	
CD31					
Negative	0	0.00%	0	0.00%	
Positive	33	100.00%	22	100.00%	
ERG					
Negative	0	0.00%	0	0.00%	
Positive	33	100.00%	22	100.00%	
CAMTA1					< 0.001*
Negative	3	8.80%	23	100.00%	
Positive	31	91.20%	0	0.00%	
TFE3					0.038*
Negative (weak, moderate)	25	73.50%	22	95.70%	
Positive (strong)	9	26.50%	1	4.30%	
Ki-67 labeling index					< 0.001*
Mean ± SD	6.0 ± 4.8		42.0 ± 20.1		
(range)	(0.1–15.7 )		(12.6–69.5 )		
< 10%	26	76.50%	0	0.00%	
≥ 10%	8	23.50%	23	100.00%	
P53 expression					< 0.001*
Aberrant pattern	1	2.90%	20	87.00%	
Wild pattern	33	97.10%	3	13.00%	

* *p*-value < 0.05; EHE: epithelioid hemangioendothelioma; AS: angiosarcoma

### Prognostic factors in EHE and survival analysis (LG EHE, vs. HG EHE, vs. AS)

To predict the prognosis of EHE, we analyzed histological and immunohistochemical findings associated with overall survival (OS) using the Cox proportional hazards model in the EHE group (Table 4). In this univariate survival analysis, mitotic activity (cut-off: 2/10HPFs, *p* = 0.035) and the Ki-67 index (cut-off: 10%, *p* = 0.021) were significantly associated with OS. Other histological findings, including necrosis, solid/sheet growth, myxohyaline stroma, sinusoidal infiltration, hypercellularity, buds, hobnails or papillary-like projections, high-grade nuclear atypia, and intracytoplasmic vacuoles were not associated with survival. Immunohistochemical findings, including TFE3 positivity, and aberrant p53 expression were also not correlated with OS.

**Table 4:** Univariate analysis of the pathologic features affecting the overall survival of patients with EHE

Variable	Category	Univariate OS analysis		
		HR	95% CI	*p*-value
Necrosis	Present	0.638	0.112–3.647	0.614
Solid/sheet growth	Present	2.049	0.091–46.034	0.651
Myxohyaline stroma	Present	0.142	0.016–1.270	0.081
Sinusoidal infiltration	Present	0.894	0.173–4.614	0.894
Hypercellularity	Present		Not estimated	
Buds, hobnails, or papillary-like projection	Present	0.414	0.076–2.262	0.309
High grade nuclear atypia	Present	0.938	0.234–3.757	0.928
Prominent nucleoli	Present	0.638	0.074–5.465	0.681
Cytoplasmic vacuoles	Present	0.142	0.016–1.270	0.081
Mitosis/10HPFs	≥ 2	5.598	1.127–27.799	0.035*
TFE3 group	Positive	0.287	0.035–2.368	0.246
Ki-67 proliferation index (%)	≥ 10	6.023	1.314–27.610	0.021*
P53 expression type	Aberrant	0.187	0.022–1.605	0.126
Tumor size (largest, cm)	≥ 5	4.917	0.98–24.683	0.053
Total number of tumor	≥ 2 (multiple)	2.221	0.243–20.342	0.480
Distant metastasis (extrahepatic)	Present	4.341	0.997–18.896	0.050
Recurrence	Present	5.526	0.675–45.236	0.111

* *p*-value < 0.05, OS: overall survival; HR: hazard ratio; CI: confidence interval; EHE: epithelioid hemangioendothelioma

Using these prognostic factors including mitotic activity and Ki-67 index, EHEs can be classified as LG EHE and HG EHE. HG EHE was defined when mitotic activity was more than 2/10HPFs or the Ki-67 index was more than 10%. As shown in Table 5, the sensitivity and specificity of mitotic grading in EHEs were 0.72 and 1.00, respectively. Those of the Ki-67 index were 0.44 and 1.00, respectively. These two diagnostic criteria can be used in the differential diagnosis between EHE and AS. The mitotic count and Ki-67 index showed high sensitivity (0.96 and 1.0, each) and specificity (0.62 and 0.76, each) in diagnosing AS. Aberrant p53 expressions also manifest with high sensitivity and specificity (0.87 and 0.97, respectively). Using these prognostic values including mitotic activity, the Ki-67 index, and aberrant p53 expression, we can classify three groups as following: LG EHE, HG EHE, and AS.

**Table 5:** Sensitivity, specificity and area under the ROC curve scores for each diagnostic and prognostic marker differentiating between EHE and AS

Score	1	1	1	2	2	2	3
Parameters	Mitosis ≥ 2/10HPFs	Ki-67 ≥ 10%	TP53 mutation	Mitosis ≥ 2/10HPFs And Ki-67 ≥ 10%	Mitosis ≥ 2/10HPFs And aberrant p53	Ki-67 ≥ 10% and aberrant p53	Mitosis ≥ 2/10HPFs, Ki-67 ≥ 10%, and aberrant p53
Sensitivity (%)	96	100	87	88	76	80	60
Specificity (%)	62	76	97	91	100	100	91
Area under ROC curve	0.787 (0.668–0.906)	0.882 (0.792–0.973)	0.920* (0.832–1.000)	0.934* (0.860–1.000)	0.913 (0.819–1.000)	0.935* (0.852–1.000)	0.913 (0.819–1.000)

*more diagnostic

EHE: epithelioid hemangioendothelioma; AS: angiosarcoma; ROC: receiver operating characteristic

**Fig. 3:**
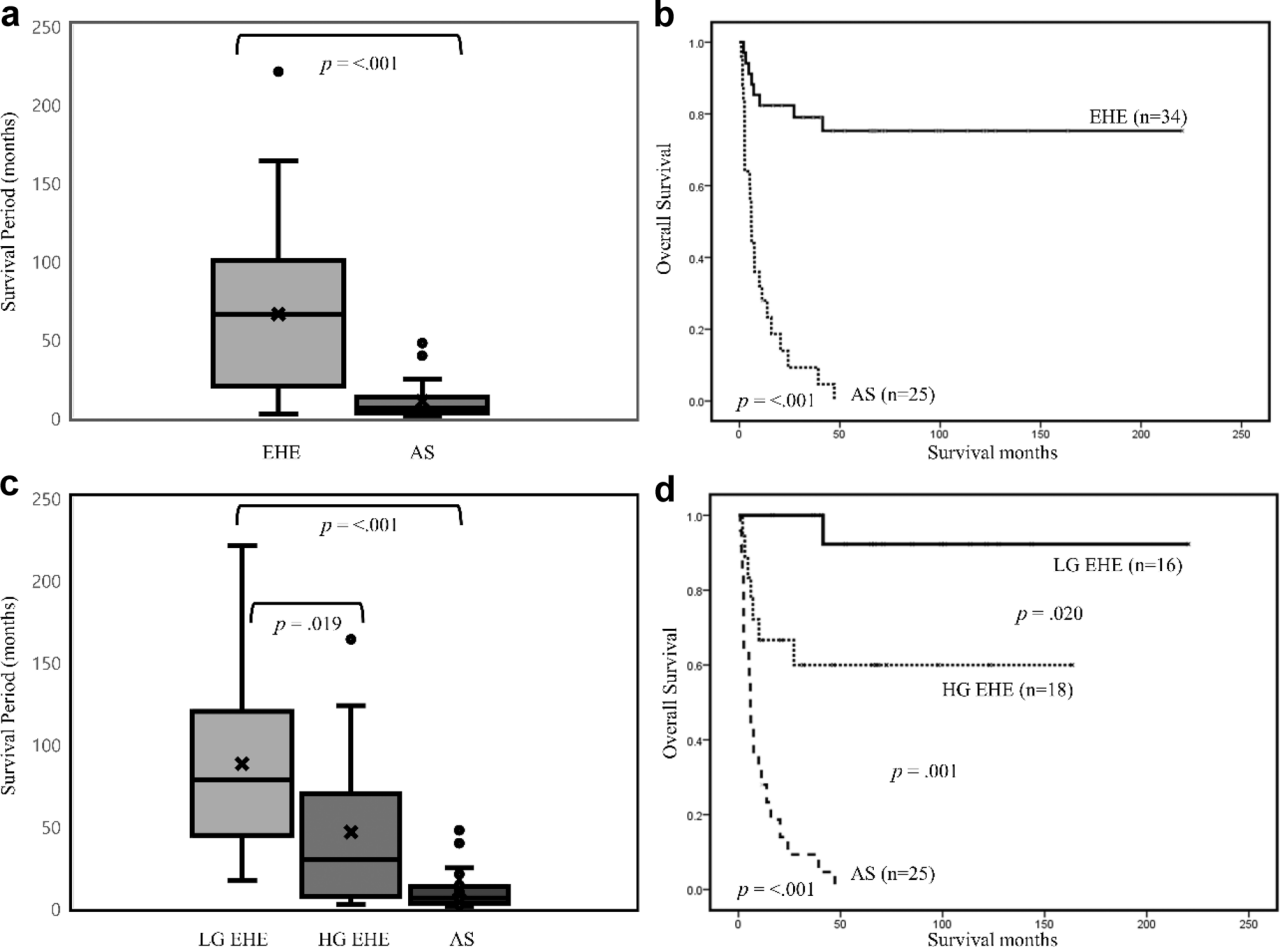
Survival analysis in LG EHE, HG EHE, and AS groups. (**A**) Patients with EHE were alive longer than those with AS (median 169.4 vs. 10.2 months, *p* = < 0.001). (**B**) In Kaplan–Meier survival analysis, EHE and AS showed significant differences in their overall survival rates (*p* = < 0.001). (**C**) Patients with LG EHE were alive longer than those with HG EHE (median 206.6 vs. 101.7 months, *p* = 0.019) and AS (median 10.2 months, *p* = < 0.001). (**D**) Three groups, including LG EHE, HG EHE and AS, showed significant differences in their survival rates (LG EHE vs. HG EHE, *p* = 0.020, LG EHE vs. AS, *p* = 0.001). EHE: epithelioid hemangioendothelioma; AS: angiosarcoma; LG: low grade; HG: high grade

In survival analysis by means of the Kaplan–Meier method, EHE and AS showed significant differences in overall survival (Fig. 3). A total of 33 patients died. Of these 33 patients, one was LG EHE, and seven were HG EHE. Most of them (25/33) were AS. Patients with EHE lived longer (median 169.4 months) than those with AS (median 10.2 months, *p* = < 0.001). The difference in survival between the two groups was significant (*p* = < 0.001). Also, the three groups, comprising the LG EHE, HG EHE and AS, showed significant differences in survival (LG EHE vs. HG EHE, *p* = .020, LG EHE vs. AS, *p* = 0.001). Patients with LG EHE lived longer than those with HG EHE (median 206.6 vs. 101.7 months, *p* = 0.019) and AS (median 206.6 vs. 10.2 months, *p* = < 0.001).

## Discussion

Malignant hepatic vascular neoplasms, including angiosarcoma and epithelioid hemangioendothelioma, are extremely rare. Although hepatic AS is well known as a rare but highly aggressive neoplasm characterized by high recurrence rates and tumor-related death, hepatic EHE can be considered clinically unpredictable because it frequently exhibits indolent behavior but sometimes develops into advanced neoplasms [1, 2, 13, 14]. The study of the pathology and radiology of primary malignant hepatic vascular neoplasms requires precise diagnosis and also the improvement of prognostic evaluation.

Compared with AS, hepatic EHE revealed a relatively well-defined border, myxohyaline stroma, buds, hobnails, papillary-like projections and cytoplasmic vacuoles. They exhibited little hemorrhaging, solid/sheet growth, and hypercellularity. In addition, the mitotic count of EHE was significantly lower than that of AS. Although these pathological features showed a statistically significant difference, it was difficult, in some cases, to accurately differentiate the two diseases based on the H&E findings alone because of overlapping features between EHE and AS. This is especially difficult when there is an abnormal morphology or when biopsy material is limited. However, immunohistochemical staining was helpful in these cases.

All hepatic EHE and AS cases included in this study were positive for endothelial markers CD31 or ERG staining. CAMTA-1 nuclear positivity was observed in 91% of EHE, and none of the AS cases was positive. A recent study by Doyle et al. [15] evaluated CAMTA-1 expression in a large series of EHE and other soft tissue neoplasms. Nuclear expression of CAMTA-1 was a highly sensitive and specific marker for EHE, observed in 86% of the total cases. It was explained by the identification of repetitive translocations involving chromosomal regions 1p36.3 and 3q25 in EHE, resulting in the formation of a fusion between *WWTR1* (WW domain-containing transcription regulator) and *CAMTA1* (calmodulin-binding transcription activator 1) [11, 15]. In published studies, the detection frequency of this fusion gene has been reported to vary (range: 77–100%) [16–20]; and the overall prevalence of this fusion gene in EHE is approximately 90%. This result was similar to that of our study. Also, more recently, a small subset of EHE was found to have an alternative *YAP1-TFE3* gene fusion [20–22]. In EHE with this fusion, the immunohistochemical results showed nuclear TFE3 was uniformly expressed, whereas CAMTA1 was negative in most cases [22]. Although the number of cases is small, our study results showed that most CAMTA1-negative cases (2 of 3, 67%) showed strong TFE3 positivity. However, it is not recommended that TFE3 immunostaining be performed alone to confirm a TFE3 rearrangement as TFE3 expression has been shown to be non-specific as confirmed in *WWTR1-CAMTA1* EHE [22]. In our study, immunostaining was performed without genetic testing. Therefore, we can only estimate the type and frequency of EHE according to histological characteristics and immunohistochemical results. In addition, significant differences were observed between the EHE and AS groups not only in CAMTA1 and TFE3 but also in the Ki-67 proliferation index and P53 expression type. The Ki-67 proliferation index was observed in more than 10% of the AS cases, and except for three cases, p53 immunostaining exhibited an aberrant pattern. Accordingly, EHE and AS could be more accurately differentiated using H&E findings as well as the immunohistochemical results of CAMTA1, P53 expression pattern, and Ki-67 proliferation index.

In previous literature, it has been mentioned that EHE has a variable clinical course [2, 14, 22–26]. However, there are still no internationally recognized pathological criteria for the prediction of EHE behavior associated with various clinical courses. Therefore, among the imaging and pathological factors, statistical analysis was performed on the factors affecting the overall survival. Mitotic activity and Ki-67 proliferation index showed significant results, and accordingly, EHE could be classified into LG and HG group. When survival analysis was performed by dividing participants into three categories: LG EHE, HG EHE, and AS, a significant graph was drawn for each group. Therefore, Ki-67 proliferation index and mitotic activity can be suggested as tools to predict the behavior of EHE. To the best of our knowledge, this is the first study to present the criteria for predicting the behavior of EHE.

**Fig. 4:**
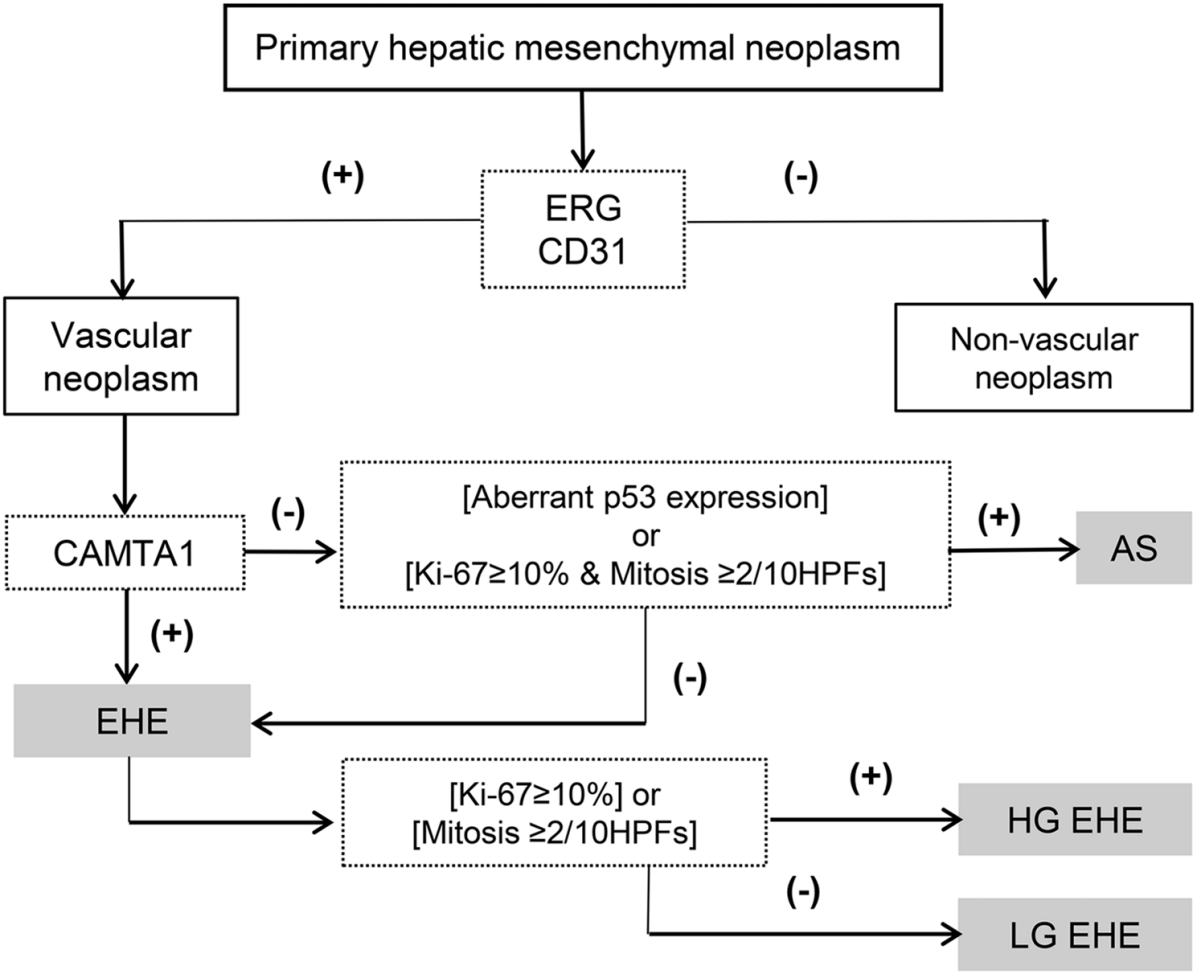
The diagnostic algorithm for primary malignant hepatic vascular neoplasms

Based on the previously mentioned results, we would like to propose the following diagnostic algorithm for primary hepatic vascular neoplasm (Fig. 4). ERG or CD31 expression confirms the vascular nature of tumor cells. CAMTA1 positivity is highly specific for the diagnosis of EHE, as none of other tumor cells reacted to this antibody. Among the CAMTA1-negative cases, if an aberrant P53 expression is identified (sensitivity 87%, specificity 97%) or the mitotic activity and Ki-67 are high (sensitivity 88%, specificity 91%), AS can be diagnosed. When less than one of the three factors, comprising mitotic activity, a Ki-67 proliferation index, and P53, are shown to be present, EHE may be diagnosed. It may be further divided into high grade and low grade according to the mitotic activity and Ki-67 proliferation index level, and accordingly, EHE behavior can be reflected and diagnosed.

Our study should be interpreted within its limitations. First, since it is not a study including a large population, further studies are needed. Second, this study was conducted retrospectively. We tried to maintain as much objectivity as possible, but prejudices that we did not consider may be involved. Finally, we have previously mentioned the types of EHE based on gene rearrangement. However, in this study, we inferred the type only from the results of CAMTA1 and TFE3 immunostaining. Hence, we were unable to evaluate the accuracy of the information on gene rearrangement because a gene study was not conducted. If a gene study is included in a following study, it is expected that the understanding of EHE will be broadened.

Immunohistochemistry for CAMTA-1, P53, and Ki-67 labeling may help distinguish EHE and AS in histologically ambiguous cases, especially in small biopsied tissue. Moreover, the combination of mitotic activity and ki-67 labeling can be a prognostic factor for EHE with various clinical behaviors.
